# Entomological survey to determine the role of cisterns in the production of *Aedes aegypti* in the U.S. Virgin Islands

**DOI:** 10.1371/journal.pone.0329222

**Published:** 2025-09-18

**Authors:** Marianyoly Ortiz-Ortiz, Rafael Saavedra-Hernández, Krystal R. Seger, Ryan Hemme, Nicole Marrero-Vázquez, Amaury Morales-González, Warren Ortiz-Ortiz, Tatiana Ortiz-Ortiz, Eduardo Burgos, Roberto Barrera, Grayson Brown, Brett Ellis

**Affiliations:** 1 Puerto Rico Science, Technology and Research Trust, San Juan, Puerto Rico; 2 Department of Health, Christiansted, United States Virgin Islands; 3 Dengue Branch, Centers for Disease Control and Prevention, San Juan, Puerto Rico; 4 Abexus LLC, San Juan, Puerto Rico; University of Maryland, UNITED STATES OF AMERICA

## Abstract

Given the limited potable water supply in the U.S. Virgin Islands, most residents use cisterns to collect rainwater and store it for their daily needs. A survey conducted in 2019 found that 45.7% of the cisterns contained mosquitoes, and 83.3% of the mosquitoes collected were *Aedes aegypti*, suggesting an important role as mosquito larval development sites. A subsequent entomological survey was designed to determine the importance of cisterns in producing *Ae. aegypti* mosquitoes and to understand the cistern factors and characteristics that influence productivity. Three floating funnel traps were installed inside each sampled cistern to collect immature mosquitoes, and exit traps were installed on the intake spouts and overflow pipes, when possible, to collect adult mosquitoes. Physical and chemical characteristics were also recorded. Yard and outdoor patio inspections were conducted at participating households to identify other types of containers with immature mosquitoes. A total of 1,858 households were visited, of which 24% granted access to their cisterns for this study. Of these, 76% of cisterns met protocol criteria, which resulted in 342 cisterns being sampled. Approximately half of the cisterns surveyed were positive for immature mosquitoes. A higher percentage was observed on the St. Thomas and St. John islands (STT District, 57.3%) than St. Croix (STX District, 40.9%). Most immature mosquitoes collected were *Ae. aegypti* (89.2%), followed by *Culex* spp*.* (1.3%), and *Ae. mediovittatus* (0.38%). Pupal surveys revealed that cisterns were the second highest contributor to the production of *Ae. aegypti* pupae, with 16.9% of the pupae collected from cisterns. However, this number might be underestimated given sampling limitations. Buckets were the highest *Ae. aegypti* pupal producer with 47.3%. On average, 5.8% of the exit traps installed on cisterns captured adult mosquitoes, with higher rates of capture on the STT district than on the STX (6.4% versus 5.3%, respectively). Most adult mosquitoes collected (90.7%) were identified as *Ae. aegypti,* while the other 9.2% were *Culex* spp*.* We can conclude that cisterns are important larval development sites for *Ae. aegypti* and vector management strategies must be developed to reduce their impact.

## Introduction

The Pan American Health Organization (PAHO) estimates that approximately 500 million people in the Americas are at risk of dengue, and the incidence has increased from 1.5 million cases in the 1980s to 16.2 million in the past decade [1]. In the first half of 2024 alone, over 9 million dengue cases were reported in the region, making it the worst year on record for the Americas [2]. Dengue virus is endemic in the Caribbean, with PAHO reporting more than 140,000 cases in the past 5 years [1]. Other vector-borne viruses such as Zika, chikungunya, and yellow fever are also prevalent in the region. The 2014 chikungunya outbreak resulted in more than 982,000 cases in the Caribbean, while the 2016 Zika epidemic affected over 153,000 people. Although reported cases have declined in recent years these diseases are still significant in the Americas, with 273,685 cases of chikungunya and 40,249 cases of Zika reported in 2022 by PAHO [3,4].

These arboviruses are transmitted mainly by *Aedes aegypti* (L.)*,* a peridomestic mosquito that is well-established in the United States Virgin Islands (USVI), a U.S. territory located in the Caribbean Sea. During a 2014 entomological survey, *Ae. aegypti* accounted for 29.7% of the adult mosquitoes collected, with the highest abundances observed on St. John and St. Thomas (21.0 and 11.0 mosquitoes/trap per day, respectively), compared to St. Croix, where the abundance was lower (3.0 mosquitoes/trap per day) [5]. The survey also revealed that a high number of homes had containers that could serve as mosquito larval habitats. Insecticide resistance testing identified elevated levels of resistance to malathion and permethrin in several local populations of *Ae. aegypti* on St. Croix Island [5].

Multiple outbreaks have been associated with *Ae. aegypti-*borne viruses in the USVI, such as Zika, chikungunya, and dengue [6–9]. Following the 2016 Zika outbreak and the 2017 hurricanes Irma and María, Seger et al. 2019 [10] conducted a series of studies that found USVI residents were highly concerned about contracting mosquito-borne diseases. Furthermore, post-hurricane damage led to the loss of protective infrastructure such as undamaged door and window screens and reduced use of air conditioning, increasing the population’s exposure to mosquito bites [10].

According to the 2020 census, most of the USVI’s population lives on three islands, St. Croix (Pop. 41,004), St. Thomas (Pop. 42,261), and St. John (Pop. 3,881), with a total estimated population of 87,146 [11]. Due to limited access to potable freshwater and the absence of a comprehensive municipal water system, it is legally mandated that most residences and buildings be equipped with cisterns for water storage. These cisterns, which function as rainwater catchment reservoirs, are typically subterranean with an average water storage capacity of 50,000 liters [12].

An entomological survey conducted in July/August of 2019 found 45.7% of the cisterns contained adult or immature mosquitoes. Immatures (larvae and pupae) were collected from 22.6% of the cisterns, while adults were collected from 40.2%. The proportion of cisterns containing mosquitoes varied across the islands, and most of the collected mosquitoes were identified as *Ae. aegypti* (83.3%) [12]. While this study demonstrated that cisterns can harbor *Ae. aegypti*, their methods had key limitations, including reliance on aspiration of accessible surfaces and net collection of visible immatures, likely underestimating mosquito abundance. To address these gaps, the present study employed standardized, systematic sampling to quantify immature mosquito production and assess how cistern characteristics influence mosquito presence. We hypothesize that (1) cisterns serve as significant larval development sites for *Ae. aegypti*, and (2) cisterns with specific features, such as infrequent maintenance or favorable organic content, are more likely to support active larval development. By testing these hypotheses, this study aims to more precisely define the role of cisterns in *Ae. aegypti* development and guide targeted vector control strategies. This is particularly important because cisterns are often located beneath homes with interior hatches, potentially allowing mosquitoes direct access into living spaces and increasing the risk of arboviral disease transmission.

## Materials and methods

### Funnel trap design and sample method validation

**Fig 1:**
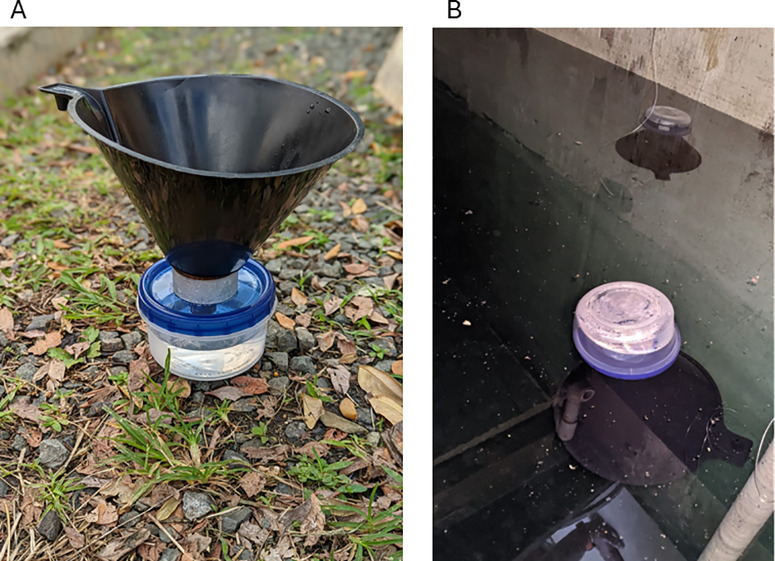
Picture of the funnel trap used in this study. It follows the design described in Burke et al. 2010 [13]. (A) shows a side view of the trap while (B) shows the traps immersed in a cistern.

Funnel traps have previously been used in Puerto Rico to sample septic tanks and demonstrate the presence of larvae in sewage water [13]. For this study, we built over 150 modified Vietraps [14] following the design described by Burke et al. 2010 [13] but with larger dimensions (funnel diameter of 20 cm, minimum draft of 19 cm and overall height of 23 cm; Fig 1).

We conducted an initial study involving 23 cisterns in St. Croix, USVI, to evaluate the performance of the sampling design for water cisterns. Households were selected through convenience sampling based on residents’ availability and willingness to participate. To determine the appropriate number of traps required for collecting immature mosquitoes, traps were deployed in sets of three, four, or five per cistern and collected after 48 hours. Five cisterns were monitored for up to six days, with samples collected every two days to assess the temporal effectiveness of the trapping method. The decision to use multiple traps per cistern was informed by prior field experience with trap deployment in septic tanks, as described by Burke et al. [13].

To facilitate retrieval, traps were secured using monofilament fishing line tied to pipes, nails, or other available fixtures near the cistern’s opening. The line length varied depending on the cistern depth and water volume. No significant differences were found when using more traps or for longer periods of time. Therefore, the use of three traps and 48-hour retrieval periods was selected as the most logistically feasible approach for full-scale implementation.

### Full study site and sample selection

The entomological survey was conducted from February 1^st^ to May 28^th^, 2021, in two geographically distinct districts: St. Croix (STX) and St. Thomas/St. John (STT). A two-stage cluster sampling scheme was employed, following the Community Assessment for Public Health Emergency Response (CASPER) methodology [15]. Using ArcGIS Portal (GIS software, Version 10.9, Redlands, CA: Environmental Systems Research Institute, Inc., 2010) and 2010 census data [8], 30 census blocks were randomly selected proportional to the estimated population. For the STT district, 28 clusters were selected on the island of St. Thomas and 2 clusters on the island of St. John. As per the CASPER methodology, the secondary sampling units (SSU) were selected using a systematic sampling approach. Once the 30 clusters were selected for each district, maps with geographical demarcations were generated. The total number of households within each cluster was estimated using the structure layer of the maps, and a random starting point was assigned. A systematic value (K) for each cluster was assigned to determine the seven households to be selected for each cluster. The CASPER methodology does not recommend identifying households before arriving at the cluster because of possible discrepancies between aerial maps and ground conditions. However, given the COVID-19 pandemic and to reduce the probability of convenience and/or targeted sampling, the procedure was conducted *a priori*.

All initially selected households were visited. If there was no response, a flyer with project details and contact information was left. Initially, households with no response were visited three times before replacing them, as recommended by CASPER. However, due to significant delays, this was adjusted after eight weeks to conduct only a second visit for non-responders before replacing them. A household was replaced if: (1) there was no response after 2 or 3 visits, (2) the structure was vacant, (3) the resident declined participation, (4) the house lacked a cistern or access to one, (5) the cistern was empty, or (6) the structure was not a household but a commercial establishment. If any of these conditions were met, the interview team went to the next household following a serpentine pattern within the cluster until identifying an available household that met the inclusion criteria.

### Sample collection and processing

Interview teams of at least two people visited each household, conducted yard and outdoor patio inspections, deployed traps in the cisterns, and completed the resident’s survey. ArcGIS applications, specifically Survey 123 and Collector (GIS software, Version 10.9, Redlands, CA: Environmental Systems Research Institute, Inc., 2010), were used to collect data on cistern characteristics, residents’ cistern maintenance practices, and cistern water parameters. A written consent form was provided to each resident to gain access to the properties.

Three funnel traps were deployed in each of the cisterns as described above and retrieved after 48 hours. The contents of each funnel trap were transferred into 18 oz. Whirl-Pak® bags and transported in coolers with icepacks. In the laboratory, immature mosquitoes were transferred into 1.5 ml Eppendorf tubes, killed, and preserved in 70% isopropyl alcohol until they were shipped to Puerto Rico for identification within 72 hours. Isopropyl alcohol was removed before shipping to comply with air cargo regulations.

**Fig 2:**
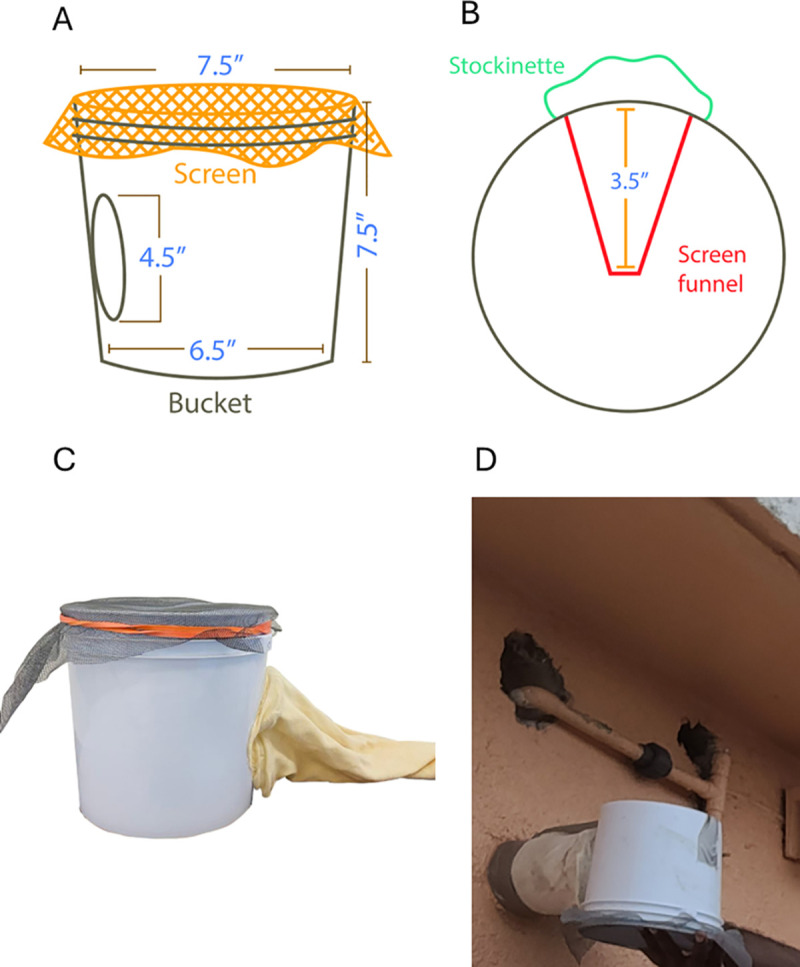
Exit traps used to capture adult mosquitoes exiting the cisterns through intake or overflow pipes. (A) shows the exterior of the trap covered with screen cloth to trap the adult mosquitoes. (B) shows a top view of the trap showing an internal funnel made using a metallic screen and a stockinette on the outside that was placed around the opening of the pipes. (C) shows a side-view picture of the trap, and (D) shows an exit trap installed in an overflow pipe.

Adults emerging from the cisterns via intake spouts and overflow pipes were collected using exit traps (Fig 2). When possible, these traps were installed on the outside of intake spouts and overflow pipes and retrieved after 48 hours. It was not possible to install traps in all spouts and pipes, as some were not accessible (e.g., too high, unsafe location or blocked). Traps with adult mosquitoes were taken to the laboratory, where the mosquitoes were killed by freezing. Dead mosquitoes were removed from the traps, wrapped in 15.2 cm x 27.9 cm paper towels, and sent to Puerto Rico for identification within 72 hours. The use of this non-alcohol technique facilitated air transport in small aircraft and was made possible by the very low species diversity of USVI mosquito fauna. In fact, *Ae. aegypti* and *Cx. quinquefasciatus* make up 98% of the mosquitoes found in the USVI [5]. Thus, the *Ae. aegypti* were still easily identifiable.

### Cistern characteristics and water properties

A laser distance meter (Bosch BLAZE Model GLM20, Robert Bosch LLC, Farmington Hills, MI) was used to measure water level, cistern height, depth, and length. Cistern type, location, and construction materials were visually noted. Water samples were collected during the first visit and tested using a Hach Pocket Pro + Multi 2 Tester (Hach Co., Loveland, CO) to measure conductivity, pH, total dissolved solids (TDS), salinity, and temperature. Probes were calibrated weekly or as needed. The total chlorine and free chlorine were measured using a Hach Pocket Colorimeter II (Cat. No. 58700−00), and turbidity was measured with a Hach 2100Q Portable Turbidimeter (Cat. No. 2100Q01).

### Yard and outdoor patio inspections for larval habitats

The yard and surrounding outdoor areas of each participant’s household were inspected for containers and other potential mosquito larval habitats. Aquatic habitats were first categorized as natural or artificial and then as wet or dry. Wet habitats were further assessed and classified as positive or negative for the presence of immature mosquitoes. Pupae found in containers were collected using a turkey baster or dipper and transferred to a Whirl-Pak® bag. In most cases, all the pupae present in each container were collected. However, approximately 15% of containers were too large or otherwise impractical to sample all pupae. Samples were processed in the laboratory following the procedures described above.

### Data analysis

Data were collected using ArcGIS Survey 123, and data analysis was performed using the Python programming language [16]. Descriptive analyses included calculating the frequency of cisterns and containers that were positive for mosquitoes. These frequencies were used to describe variables of interest, including household and cistern characteristics, water parameters, and mosquito presence and abundance in cisterns versus other containers.

The Breteau Index (number of containers with any larvae or pupae, divided by the total number of inspected households, multiplied by 100) and pupae per household index (total number of pupae found, divided by the total number of inspected households) were calculated for each district. The container index (percentage of water-holding containers infested with larvae and pupae out of those inspected) was calculated for each household. A t-test was conducted to compare container indices for the two districts. Indices were calculated using only data from containers other than cisterns.

A correlation analysis was performed to assess associations between cistern water parameters and the presence or absence of immature mosquitoes. To estimate the adult mosquito production, the “relative importance” of each type of container was calculated by dividing the total number of pupae found in each container category by the total number of pupae found in all containers within the study area [17,18]. Manrique-Saide et al. 2011 define relative importance as the proportion of pupae produced by each container type at a given time, serving as a proxy for estimating adult mosquito production [18].

### Ethics statement

This study was reviewed and approved by the Ponce Medical School Foundation, Inc. Institutional Review Board, Protocol 2101051509 Exempt. Participants provided written consent before allowing access to their property.

## Results

### Cisterns and water characteristics

**Fig 3:**
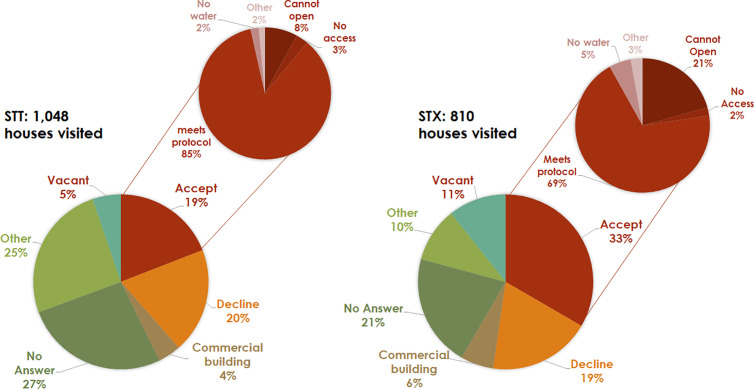
Distribution of households visited by category. The larger pie charts show the total number of households visited. Of the 19% in the STT district and 33% in the STX district that accepted to participate (smaller pie charts), 85% in the STT and 69% in the STX met protocol criteria to be included in the survey. Cisterns classified as “Other” include cisterns that were too old or damaged, access was too small, hard to coordinate a visit, among others.

A total of 1,858 households were visited, and consent was requested to inspect the water cisterns and conduct yard and outdoor patio inspections. Consent was obtained for 447 (24%) of the households visited. The acceptance rate was higher in the STX district than in the STT district; however, the proportion of consenting households that met protocol criteria was higher in STT than in STX (Fig 3). Of the households that met the criteria, 171 were fully inspected in each district for a total of 342 households. This represented 81.4% of the target sample of 420 households. According to the CASPER methodology, achieving at least 80% of the target sample size is sufficient for valid data analysis and study success. Most fully inspected households were single-family units. However, in the STT district, more multiple-family units were inspected than in the STX district (Table 1). Most of the cisterns inspected were made of concrete, located underground) and had a square or rectangular shape (Table 2).

**Table 1:** Distribution of the type of households that participated in the survey.

Type of household	STT (%)	STX (%)	Total (%)
Single-family	118 (69.0)	155 (90.6)	273 (79.8)
Multifamily	43 (25.1)	8 (4.7)	51 (14.9)
Apartment	9 (5.3)	3 (1.8)	12 (3.5)
Other	1(0.6)	4 (2.3)	5 (1.5)
**Total**	**171 (50.1)**	**170 (49.9)**	**341 (100)**

**Table 2:** Distribution of the type of cisterns inspected. A total of 342 cisterns were inspected, 171 in the STT district and 170 in the STX district.

Type of cistern	STT (%)	STX (%)	Total (%)
Above ground	31 (18.1)	25 (15.2)	57 (16.7)
Underground	140 (81.9)	145 (84.8)	285 (83.3)
Not under the house	27 (15.8)	31 (18.1)	58 (17.0)
Under the house	144 (84.2)	139 (81.9)	284 (83.0)
Circle	12 (7.0)	8 (4.7)	20 (5.8)
Rectangle/Square	159 (93.0)	162 (95.3)	322 (94.2)
Concrete	159 (93)	163 (95.9)	323 (94.4)
Plastic	12 (7.0)	7 (4.1)	19 (5.6)

Water samples collected directly below the cistern entrance showed an average water temperature of 28.04°C ± 0.18, which falls within the ideal range for *Ae. aegypti* larval development [19]. No significant differences were found between the STX and STT cistern water temperatures, nor in the other measured water parameters, including pH, salinity, conductivity, TDS, turbidity, free chlorine, and total chlorine (Table 3). Similarly, there were no significant differences in water parameters between cisterns with immature mosquitoes present and those without.

**Table 3:** Mean and range of water parameters grouped by cisterns with and without immature mosquitoes. The values for cisterns with and without immatures were compared, and no significant differences were found.

Parameter	Cisterns with immatures				Cisterns without immatures				Degrees of freedom
	N	Mean	Max	Min	N	Mean	Max	Min	
Temperature (°C)	179	27.8	33.0	23.7	153	28.0	35.8	23.7	294
pH	185	8.2	11.4	6.1	154	8.2	10.3	5.1	313
Conductivity (µS/cm)	186	203.2	1320.0	36.2	154	252.2	2610.0	50.8	247
Salinity (ppt)	187	0.1	0.7	0.0	154	0.1	1.4	0.0	243
Total Dissolved Solids (ppm)	187	158.2	938.0	26.2	154	142.8	1080.0	1.0	334
Turbidity (NTU)	187	1.6	29.2	0.2	154	2.3	60.8	0.2	212
Free Chlorine (mg/L Cl2)	187	0.1	0.9	0.0	154	0.1	2.2	0.0	182
Total Chlorine (mg/L Cl2)	187	0.1	1.2	0.0	154	0.2	2.2	0.0	245

### Mosquito presence and abundance

Mosquito immatures were found in 188 (56.6%) cisterns with a mean of 22.71 (SE + /- 5.31) immatures per cistern. A Chi-square analysis found a significantly higher number of positive cisterns for immatures in STT (64.3%) than in STX (45.8%). Most mosquitoes captured by the funnel traps were in the larval stage, although 40 (12%) cisterns, 22 in STT, and 18 in STX, also had pupae present. No significant correlations were found between the presence or absence of immatures and the cisterns’ physical characteristics, residents’ cistern maintenance practices, or cistern water parameters.

Adults were captured by the exit traps in 23 (6.9%) of the cisterns sampled. Of these, 19 were also positive for immatures, from which 10 were also positive for pupae. Adult predators were found in 22 of the 647 (3.4%) exit traps installed. Most of these were frogs, which could have reduced the number of positive exit traps. Although adult mosquitoes were found in exit traps on both intake spouts and overflow pipes, it was more common to find adults in exit traps on overflow pipes (80.6%). The maximum number of adults from a single exit trap was 15 from STT; all were *Ae. aegypti,* and no pupae were captured from that cistern.

Immature *Ae. aegypti* mosquitoes were found in 175 of the 188 cisterns positive for immatures. The majority of immature mosquitoes caught were identified as *Ae. Aegypti* (Table 4). Similarly, adults captured were also mostly *Ae. aegypti*, with 56.9% identified as females.

**Table 4:** Species distribution by life stage of the specimens collected using funnel traps from all cisterns. Adults were captured using exit traps.

Stage	Species	STT (%)	STX (%)	Total (%)
***Immatures*** ^*^	*Ae. aegypti*	2891 (90.3)	1001 (86.1)	3,892 (89.2)
	*Ae. mediovittatus*	15 (0.468)	1 (0.086)	16 (0.367)
	*Cx. quinquefasciatus*	2 (0.062)	22 (1.89)	24 (0.55)
	*Culex* spp.	6 (0.188)	11 (0.947)	17 (0.39)
	*Cx. nigripalpus*	3 (0.093)	14 (1.20)	17 (0.39)
	Unclassified	283 (8.84)	113 (9.72)	396 (9.08)
	***Total Immatures***	**3,200 (100)**	**1,162 (100)**	**4,362 (100)**
***Pupae***	*Ae. aegypti*	59 (95.2)	50 (96.2)	109 (95.6)
	*Culex* spp.	3 (4.84)	2 (3.85)	5 (4.39)
	***Total pupae***	**62 (100)**	**52 (100)**	**114 (100)**
***Adults***	*Ae. aegypti*	36 (90)	23 (92)	59 (90.8)
	*Culex* spp.	2 (5)	0 (0)	2 (3.08)
	*Cx. quinquefasciatus*	2 (5)	2 (8)	4 (6.15)
	**Total Adults**	**40 (100)**	**25 (100)**	**65 (100)**

* Immatures include both larvae and pupae.

### Yard and outdoor patio inspections

Yard and outdoor patio inspections for mosquito larval habitats were conducted at 335 of the 341 households that participated in the survey (168 in STT and 167 in STX). Potential larval habitats (with or without water) were found in 98.2% of the households inspected, and mosquito immatures were found in 34.9% of those. A slightly higher number of households had potential mosquito larval habitats in the STX district (99%) compared with the STT district (97%). However, 40.5% of the households in the STT district had positive larval habitats compared to 29.3% in the STX district. Of the households inspected, 63 (18%), 46 in STT and 17 in STX, had pupal presence. Among these, 50 households (79.4%), 39 in STT and 11 in STX, were positive for *Ae. aegypti* pupae.

**Fig 4:**
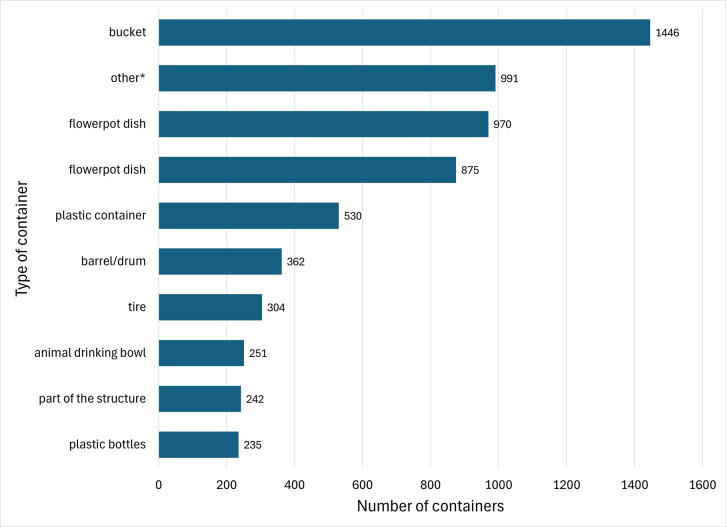
Distribution of the most common container types found during yard and outdoor patio inspections in the USVI. Buckets were the most frequently observed container type, with 1,446 items counted. The Other* category included old furniture and appliances, lids, kitchen pots, storage/toolboxes, and metal trays.

Overall, 821 water-holding containers were found in the STT district and 614 in the STX district. Of these, 57 (6.9%) and 14 (2.2%), respectively, were positive for *Ae. aegypti* pupae. Other pupal species found in containers were *Cx. biscaynensis* and *Cx. quinquefasciatus*. The most common artificial containers found were buckets, plastic containers, barrels/drums, flowerpot dishes, and flowerpots (Fig 4). Buckets, barrels/drums, and tires were the containers with the highest numbers of *Ae. aegypti* pupae found (Table 5).

**Table 5:** Number of *Ae. aegypti* pupae found in larval habitats, including cisterns, in the USVI.

Type of Container	Number of positive containers for pupae	Total number of pupae in each type of container	Contribution to the total number of *Ae. aegypti* pupae (%)*
bucket	32	305	47.3
cistern	38	109	16.9
barrel/drum	13	65	10.1
tire	3	46	7.13
trashcan	3	32	4.96
plastic container	5	24	3.72
flowerpot	3	23	3.57
discarded kitchen appliances	1	19	2.95
other	3	12	1.86
plastic bottle	1	3	0.47
plastic bottle cap	1	3	0.47
glass container	2	2	0.31
flowerpot dish	1	1	0.16
glass bottle	1	1	0.16
**Total**	**107**	**645**	**100**

*The contribution to the total number of *Ae. aegypti* pupae was calculated by dividing the total number of pupae in each type of container by the total number of pupae collected. For non-cistern containers, nearly all pupae were counted. However, the sampling method for cisterns only sampled from an average of about 0.5% of the cistern surface area.

The Breteau index and Pupae per household index were higher in STT (125.59 and 4.09, respectively) than STX (54.38 and 0.58). The container index was also calculated and compared between districts. While the average container index appeared higher in STT (9.99) than in STX (6.84), the difference was not statistically significant (p-value = 0.066). When comparing households with positive cisterns with those with positive containers, they were associated in only 62% (69/111) of the overall cases (Table 6). Thus, if a positive container was used to indicate a positive cistern at a household, nearly 40% of the positive cisterns would be unrecognized and unaddressed.

**Table 6:** Contingency table comparing the number of positive and negative cisterns with positive and negative larval habitats.

		Larval habitats		
Cisterns		Positive	Negative	Total
	Positive	69	116	185
	Negative	42	96	138
Total		111	212	323

## Discussion

This study aimed to understand if cisterns were productive larval development sites for *Ae. aegypti*, the main arbovirus vector of chikungunya, dengue, and Zika viruses in USVI. Our findings support the hypothesis that cisterns serve as significant larval development sites for *Ae. aegypti*. Mosquito larvae and pupae were found in most of the cisterns inspected, consistent with previous findings by Seger et al. 2022, where 46% of the cisterns inspected were positive for mosquitoes [12]. More importantly, our study captured pupae from cisterns, which is a reliable indicator of mosquito production [20,21]. To calculate the relative importance of each type of larval development site, we used the method described by Manrique-Saide et al. 2014 [14], which states that to assess how much a container contributes to pupal production, it is important to determine the proportion of the total number of pupae collected from that container. Applying this principle to our study, we determined cisterns contributed 16.9% of the total number of pupae collected across all container types, ranking second only to buckets (47.3%; Table 5). However, their actual contribution to pupal production is likely underestimated due to the logistical challenges in fully sampling cisterns (e.g., limited access and small surface area covered by traps). Cisterns varied in size, shape, and dimensions; therefore, it was not possible to identify a representative cistern structure to create a standard curve, as described in Russell and Kay 1999 [14]. The cisterns had an average surface area of 18.1 m^2.^ Although the traps could float inside the cistern, they only covered an area of approximately 0.093 m^2^ at a given time and likely caught only a small percentage of the total pupae inside a cistern. In contrast, we could directly count all or nearly all pupae in buckets and other containers. Thus, the consistent detection of immature stages and their proportional contribution to total pupae strongly support the conclusion that cisterns are significant larval development sites for *Ae. aegypti* in the USVI.

This study did not support the second hypothesis. Water parameters in cisterns did not vary significantly between those with or without immature mosquitoes, a finding consistent with Seger et al. 2022 [12]. All cisterns provided water conditions suitable for larval development. Temperature is one of the most critical factors influencing the survival and development of mosquito larvae [22]. In this study, cistern water temperatures ranged from 23.7°C to 35.8°C. *Ae. aegypti* has been shown to tolerate temperatures ranging from 16°C to 34°C [19,23] with optimal development occurring at 32°C [23,24]. Water pH fluctuated between 5.7 and 11.6, values within the tolerance range of *Ae. aegypti* larvae [25]. Overall, there was a lack of correlation between the cistern water parameters and the presence of immature mosquitoes (Table 4). Differences in larval populations are therefore more likely attributable to other factors such as variations in structural features of the roofs, drainage systems, and cisterns; differences in roof orientation to sunlight or shade; or other microhabitat differences. Structural vulnerabilities, such as unmaintained drainage channels and water storage systems, have been shown to influence mosquito breeding patterns [26–28], while urbanization and microclimatic differences, including shading and habitat structure, affect mosquito abundance [29,30]. In Puerto Rico, defects in septic tanks, such as cracks and uncovered access ports, were associated with higher larval presence, emphasizing the role of cistern integrity [31]. Additionally, *Ae. aegypti* females exhibit skip-oviposition behavior, distributing their eggs across multiple sites [32]. This behavior is influenced by factors such as the presence of conspecific larvae and pupae, container fill method, container size, presence of a lid, and sun exposure [33], resulting in heterogeneous larval distributions even under similar environmental conditions. Thus, structural and microhabitat differences, rather than water quality parameters alone, likely play a critical role in determining the presence and abundance of immature mosquitoes in cisterns.

One limitation of this study was the difficulty in recruiting households due to ongoing COVID-19 concerns and resident availability. Therefore, to meet the target sample size, we limited repeat visits to non-responsive households. Another limitation of this study was the inability to collect all immature mosquitoes present in the cisterns, which prevented the estimation of total abundance data. Sampling adult mosquitoes using exit traps also presented challenges, as not all intake and overflow pipes were accessible for trap placement; many were located on rooftops or other inaccessible areas. This limited the collection of all adult mosquitoes exiting the cisterns and further restricted the ability to obtain total abundance estimates. Additionally, it is possible that adult or larval mosquitoes entered the cisterns through uncovered pipes, as not all openings were sealed during the study. Nevertheless, the high presence of pupae in the cisterns indicates that cisterns served as active *Ae. aegypti* production sites rather than merely functioning as adult resting sites.

Access to cisterns is typically located indoors, requiring residents’ permission to enter the home. In many cases, accessing and opening the cistern hatch involved moving heavy furniture and floor coverings, an intrusion often perceived as burdensome by residents. As a result, periodic or long-term (e.g., annually) sampling may not be feasible, as it can discourage resident participation. Consequently, sampling from cisterns is likely to remain challenging. Ideally, it would be advantageous to predict positive cisterns based on the presence of other positive containers outside the home. However, the finding that only 62% of the households with positive containers also had positive cisterns indicates that the external containers’ status is not a reliable decision-making tool for mosquito surveillance or control efforts targeting cisterns. Another potential predictive approach could consider the short flight range of *Ae. aegypti*; one might expect adult mosquitoes to be more common near homes with actively producing cisterns. Thus, the use of adult mosquito traps, such as CDC light traps or BG-Sentinel traps, around homes could represent an additional strategy for identifying active cisterns. Nonetheless, the relationship between positive cisterns and other positive containers remains unclear and warrants further investigation.

In conclusion, this study makes a compelling case for developing a cistern-specific vector management program in the USVI. Such a program should integrate community engagement, homeowner education, outdoor container surveillance, and homeowner-led control interventions to reduce *Ae. aegypti* populations and the transmission of arboviral diseases. Work conducted by Barrera *et al*. 2008 [34] in Puerto Rico demonstrated that integrated vector management strategies can effectively reduce *Ae. aegypti* populations, and other studies have similarly shown that well-designed strategies can reduce mosquito populations and lower arboviral disease risk [35,36].

Government-supported efforts would be essential to retrofit cisterns by sealing openings, installing screened vents, and incorporating barriers like one-way valves to prevent mosquito entry while preserving water access. Resident engagement must also include training on maintaining cisterns in sealed conditions and minimizing mosquito access during water collection, cleaning, and use.

One major limitation to implementing a cistern-focused vector control program is the scarcity of approved mosquito control options for use in potable water within the United States. Although the USVI Department of Health advises against drinking cistern water, over 80% of the population relies on rainwater catchment systems for bathing, washing dishes, and drinking, particularly following natural disasters such as hurricanes [12,37]. Therefore, cistern water should be treated as potable for mosquito control interventions. Potential interventions include chlorination, acoustic controls [38], and the Special Local Needs registration of WHO-approved larvicides. While options are limited, existing methods are sufficient to implement an integrated cistern-focused mosquito control strategy. Our findings underscore the importance of such a program to reduce arboviral disease risk in the USVI.

## Supporting information

S1 File Cistern survey raw data. Includes raw data collected during the house visits and cistern inspections.
(XLSX)

S2 File Larval habitats survey raw data. Includes raw data collected during the house and patio inspections to determine the presence of larval development sites.
(XLSX)
